# The Diagnostic and Prognostic Role of Inflammatory Markers, Including the New Cumulative Inflammatory Index (IIC) and Mean Corpuscular Volume/Lymphocyte (MCVL), in Colorectal Adenocarcinoma

**DOI:** 10.3390/cancers17060990

**Published:** 2025-03-15

**Authors:** Robert-Emmanuel Șerban, Dragoș-Marian Popescu, Mihail-Virgil Boldeanu, Dan Nicolae Florescu, Mircea-Sebastian Șerbănescu, Vasile Șandru, Afrodita Panaitescu-Damian, Dragoș Forțofoiu, Rebecca-Cristiana Șerban, Florin-Liviu Gherghina, Cristin-Constantin Vere

**Affiliations:** 1 Department of Gastroenterology, University of Medicine and Pharmacy of Craiova, 200349 Craiova, Romania; drrobert.serban03@gmail.com (R.-E.Ș.); dan.florescu@umfcv.ro (D.N.F.); cristin.vere@umfcv.ro (C.-C.V.); 2 Research Center of Gastroenterology and Hepatology, University of Medicine and Pharmacy of Craiova, 200638 Craiova, Romania; 3 Doctoral School, University of Medicine and Pharmacy of Craiova, 200349 Craiova, Romania; 4 Department of Extreme Conditions Medicine, University of Medicine and Pharmacy of Craiova, 200349 Craiova, Romania; 5 Department of Immunology, University of Medicine and Pharmacy of Craiova, 200349 Craiova, Romania; 6 Department of Medical Informatics and Biostatistics, University of Medicine and Pharmacy of Craiova, 200349 Craiova, Romania; 7 Department of Gastroenterology, “Carol Davila” University of Medicine and Pharmacy, 050474 Bucharest, Romania; 8 Clinical Department of Gastroenterology, Bucharest Emergency Clinical Hospital, 014461 Bucharest, Romania; 9 Department of Internal Medicine, University of Medicine and Pharmacy of Craiova, 200349 Craiova, Romania; 10 Department of Cellular and Molecular Biology, University of Medicine and Pharmacy of Craiova, 200349 Craiova, Romania; 11 Department of Medical Rehabilitation, University of Medicine and Pharmacy of Craiova, 200349 Craiova, Romania

**Keywords:** colorectal cancer, inflammatory markers, cumulative inflammatory index—IIC, average crepuscular volume–lymphocyte ratio—MCVL

## Abstract

**Background**: Colorectal cancer affects a large number of patients worldwide, with numerous factors being involved in its etiopathogenesis and chronic inflammation playing an essential role in tumor development. In this study, we analyzed and compared several markers of inflammation that are relatively easy to obtain for a rapid and accurate diagnosis and prognosis. **Methods**: This study included 219 patients diagnosed with colorectal cancer, analyzing the inflammation scores derived from their blood cells and inflammatory circulating proteins. These inflammatory markers are neutrophil-to-lymphocyte ratio—NLR; platelet-to-lymphocyte ratio—PLR; lymphocyte-to-monocyte ratio—LMR; systemic immune inflammation index—SII; systemic inflammatory response index—SIRI; aggregate index of systemic inflammation—AISI; derived neutrophil-to-lymphocyte ratio—dNLR; C-reactive protein-to-albumin ratio—CAR; and fibrinogen-to-albumin ratio—FAR. In the analysis of patients with colorectal cancer, we have also introduced two new recently developed inflammatory markers: the cumulative inflammatory index (IIC) and the ratio between the mean corpuscular volume and lymphocytes (MCVL). This study aimed to correlate the inflammatory markers’ levels with the colorectal cancer diagnostic stage, the tumor and clinical characteristics of the colorectal cancer patients, and 36 months’ survival time and to evaluate the diagnostic and prognostic capacity and accuracy of these inflammatory markers in this type of cancer. **Results**: We showed that the levels of the analyzed inflammation markers correlate with the TNM stage, the tumor pathological differentiation grade, the age and gender of the patients, and overall survival, with their increased levels being associated with a lower survival rate. **Conclusions**: The analyzed markers, which are easy to perform right from the patient’s admission, can be helpful both in diagnosis and, mostly, in prognosis, sustaining the role of inflammation in cancer. By comparing them, we showed which one can be useful for increased sensitivity and specificity in the diagnosis and prognosis of colorectal cancer patients.

## Simple Summary

Colorectal cancer causes approximately one million deaths annually. Early diagnosis and accurate prognosis can lead to increased survival rates for patients with this type of cancer. Based on simple blood tests, such as complete blood count, numerous inflammatory markers have been studied in cancers, including colorectal cancer. In this study, we analyzed several hematological inflammation markers (NLR, PLR, LMR, dNLR, SII, SIRI, and AISI) and markers based on changes in circulating inflammatory proteins (CAR and FAR) in 219 patients with colorectal adenocarcinoma. We also analyzed two new markers, IIC and MCVL. We correlated all these inflammatory markers with different tumor and clinicopathological characteristics. We compared them to see which one may be more useful in the diagnosis and prognosis of colorectal adenocarcinoma, which has better sensitivity and specificity, and which correlates better with the survival rate of these patients.

## 1. Introduction

Colorectal cancer is one of the biggest global health problems, representing approximately 10% of deaths by cancer [1]. Multiple risk factors are associated with this type of cancer, such as a sedentary lifestyle, a diet rich in saturated fat and red meat, and low fiber [2]. Although in almost 90% of cases, the sporadic form with the adenomatous polyp-adenocarcinoma sequence is the most frequent cause in the pathogenesis of colorectal cancer, it can also appear in the evolution of inflammatory bowel disease, ulcerative colitis, and Crohn’s disease, showing that it can be associated with chronic inflammation [3,4]. Chronic inflammation is related to the development of digestive cancers, such as colorectal, liver, esophageal, and gastric cancer, primarily through cytokines, chemokines, and leukocytes [5,6,7,8,9].

Various markers of inflammation have increased levels in chronic inflammation and neoplasms: C-reactive protein, fibrinogen, erythrocyte sedimentation rate, cytokines, and leukocytes [10,11,12,13]. In inflammation, the level of neutrophils increases, having a role in the recruitment of other cells, such as macrophages and monocytes, and the number of lymphocytes decreases due to cell redistribution and apoptosis [14,15,16]. Platelet numbers increase in inflammatory diseases such as inflammatory bowel disease and rheumatoid arthritis and also in cancers, where they have a role in the promotion, development, angiogenesis, and progression of tumor cells [17,18].

Fibrinogen, C-reactive protein, and albumin are proteins synthesized mainly by the liver [19,20]. C-reactive protein is an inflammatory marker whose blood levels rise in infections, inflammatory states, injuries, and cancers [21]. The blood level of fibrinogen increases in inflammatory and hypercoagulability states and in cancers [22]. Albumin is a circulating protein indicating nutritional status and has an antioxidant and anti-inflammatory role [23,24]. In chronic inflammations and advanced cancers, albumin synthesis is lower, mainly under the influence of the pro-inflammatory cytokines IL-6 and TNFα [25].

Considering that inflammatory cells’ circulating levels change in inflammatory diseases and cancers, for diagnosis and disease prognosis, several inflammatory markers based on the ratio between two complete blood count cells that are easy to determine have been studied over time, such as neutrophil-to-lymphocyte ratio (NLR), platelet-to-lymphocyte ratio (PLR), and lymphocyte-to-monocyte ratio (LMR) [13,26,27,28]. Based on the more complex ratios between three or more complete blood count cells, other inflammatory markers have been studied in various inflammatory diseases and different types of cancers to accurately determine the changes between the innate and adaptive immune response in inflammatory states. These are the systemic immune inflammation index (SII), systemic inflammation response index (SIRI), aggregate index of systemic inflammation (AISI), and derived neutrophil to lymphocyte ratio (dNLR) [29,30,31,32,33].

The mean corpuscular volume-to-lymphocyte ratio (MCVL) and the cumulative inflammatory index (IIC) are two new hematological inflammatory markers determined by the red cell distribution width (RDW) and mean corpuscular volume (MCV) changes together with neutrophil and lymphocyte level changes and have been studied in pancreatitis and ulcerative colitis [34,35]. These new markers show us the changes in erythrocytes and leukocytes in inflammatory states, highlighting the possible interactions between blood cell levels and characteristics in inflammatory states, including cancer.

Apart from the hematological inflammatory markers, other biomarkers composed of the inflammatory circulating proteins were studied: C-reactive protein-to-albumin ratio (CAR) and fibrinogen-to-albumin ratio (FAR). These markers correlate with sepsis; with inflammatory diseases such as inflammatory bowel diseases, rheumatoid arthritis, and acute pancreatitis; and also with different types of cancers such as esophageal, pancreatic, and colorectal cancer [36,37,38,39,40,41].

We analyzed all these inflammatory markers in colorectal cancer patients and correlated and compared their levels according to their TNM stages, tumor invasion, lymph node and distant organ metastasis, and pathological differentiation grade and patient characteristics. We showed their usefulness in the diagnosis process and prognosis and that they can be used as predictive markers of overall survival in patients with colorectal cancer. We also used two new inflammatory markers, IIC and MCVL, to study new tools that can help with the diagnosis and prognosis of colorectal cancer patients.

## 2. Materials and Methods

This study was approved by the Ethics Committee of the University of Medicine and Pharmacy of Craiova, No. 4/21.01.2022.

### 2.1. Inclusion Criteria for Patients and Study Design

This retrospective study included 219 patients with colorectal cancer diagnosed at the Craiova County Emergency Clinical Hospital and the Craiova Gastroenterology and Hepatology Research Center between January 2019 and January 2021.

**Figure 1:**
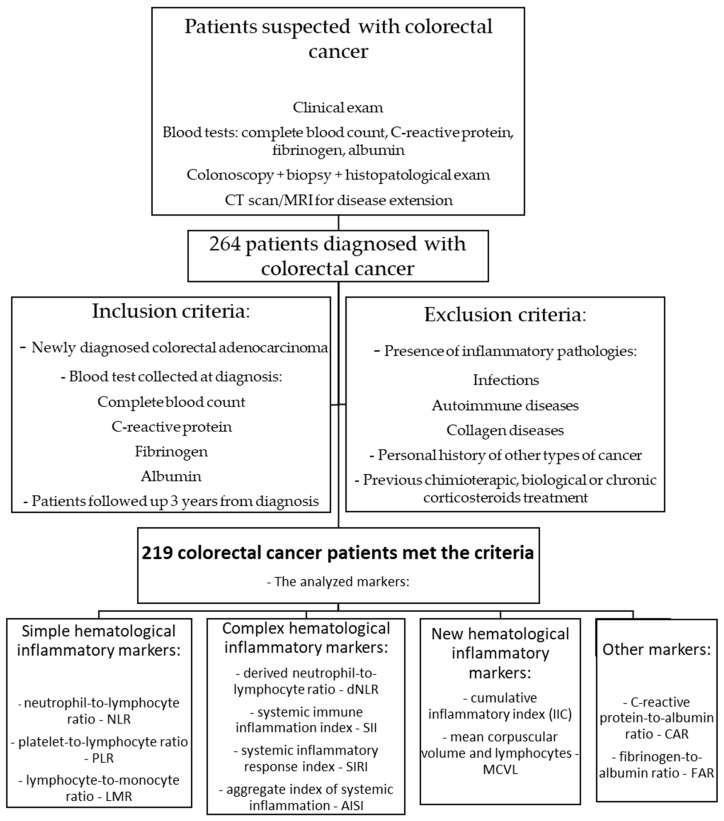
Colorectal cancer patients’ selection process.

At diagnosis, a complete clinical exam, blood tests, and a colonoscopy with biopsy, followed by histopathological diagnosis and evaluation of cancer extent by imaging (CT scan of the chest, abdomen, and pelvis or MRI for rectal cancer) for disease staging were performed in all patients. The blood tests selected for this study were represented by the complete blood count (hemoglobin, red cell distribution width (RDW-CV), mean corpuscular volume (MCV), platelets, neutrophils, lymphocytes, and monocytes), C-reactive protein, fibrinogen, and albumin (Figure 1).

The inclusion criteria were patients newly diagnosed with colorectal adenocarcinoma; patients with all the blood tests necessary to determine the inflammatory markers analyzed in this study and collected during diagnosis; and patients who could be followed for 36 months from diagnosis—this period was chosen due to the possibility of selecting patients starting during the year 2019, due to the limitations of the hospital informatics database. The exclusion criteria were the presence of pathologies that could influence inflammation markers, such as infections, autoimmune diseases, and collagen diseases; previous chemotherapy, biological, or chronic corticosteroid treatment; and a personal history of other types of cancers.

Based on the blood test results, hematological inflammation markers represented by simple or more complex ratios between complete blood count cells (neutrophils, monocytes, lymphocytes, and platelets) were determined: neutrophil-to-lymphocyte ratio (NLR), platelet-to-lymphocyte ratio (PLR), lymphocyte-to-monocyte ratio (LMR), derived neutrophil-to-lymphocyte ratio (dNLR)—neutrophils/(leucocytes–neutrophils); systemic immune inflammation index (SII)—(neutrophils–platelets)/lymphocytes; systemic inflammatory response index (SIRI)—(neutrophils–monocytes)/lymphocytes; and aggregate index of systemic inflammation (AISI)—(neutrophils–monocytes–platelets)/lymphocytes.

Two new recently studied markers, determined from the ratio between the variation of erythrocytes (MCV and RDW) and neutrophils or lymphocytes, were included in this study: cumulative inflammatory index (IIC)—mean corpuscular volume—width of erythrocyte distribution—neutrophils)/(lymphocytes—1000) and mean corpuscular volume and lymphocytes (MCVL)—mean corpuscular volumes/lymphocytes.

Other markers based on circulatory inflammatory protein level changes in inflammatory states were determined: CRP-to-albumin ratio (CAR) and fibrinogen-to-albumin ratio (FAR).

### 2.2. Statistical Analysis

For colorectal cancer patients, the levels of the inflammatory markers were analyzed for each category according to their clinicopathological and tumor characteristics (which included sex, age, primary tumor location, TNM staging, and pathological differentiation degree), and then the statistical analysis was performed.

We used Microsoft Excel 2021 (Microsoft Corp., Redmond, WA, USA), EasyMedStat (version 3.24), and SPSS 26 (IBM Corp., Armonk, NY, USA) for the statistical analysis, tables, and figures. The Shapiro–Wilk test was performed to assess the distribution of continuous data. If the data were normally distributed, we used the ANOVA test for multiple groups, followed by Tukey’s post hoc and Student’s *t*-test when two groups were compared. For the non-normal data distribution, we used the Kruskal–Wallis test for multiple groups, followed by Dunn’s post hoc test and the Mann–Whitney U test when two groups were compared. The differences were considered statistically significant at *p* < 0.05.

Receiver operating characteristic (ROC) curves were performed to analyze the sensitivity and specificity of the markers in the diagnosis and prognosis of colorectal cancer. Also, cut-off levels depending on the presence of advanced cancer were determined.

The patients were followed up for 36 months. The Kaplan–Meier curve was performed to assess the survival time in colorectal cancer patients after being divided into two categories: patients with low and high levels of each analyzed marker, depending on their cut-off level.

## 3. Results

In this study, from the 219 patients with colorectal adenocarcinoma who met the inclusion criteria, there were 127 men and 92 women. Patients were between 33 and 91 years old, with a mean age of 70. The most frequent primary tumor location was in the sigmoid, followed by the rectum, while the most uncommon was in the cecum, followed by the descending colon. Patients were classified according to the TNM classification (8th edition)—the tumor extent (T), lymph node metastasis (N), distant organ metastasis (M), and the pathological differentiation degree (G). Table 1 shows the case distribution according to clinicopathological and tumor characteristics. According to the TNM classification, most cases were diagnosed in stage II, and the fewest cases were diagnosed in stage I. According to the tumor extent, T3 tumors were the most frequent, and T1 tumors were the least frequent. According to the lymph node and distant metastasis, most cases were N0—without lymph node metastasis—and M0—without distant organ metastasis. According to the tumor pathological differentiation grade, the most common tumors were moderately differentiated, G2, followed by poorly differentiated tumors, G3, and then the well-differentiated tumors, G1.

### 3.1. Correlation of Inflammatory Markers with Clinicopathological and Tumor Characteristics in Colorectal Cancer Patients

#### 3.1.1. Correlation of Inflammatory Markers with TNM Stages

All inflammation markers were correlated with the TNM classification. All markers had increasing levels with stage advancement, except LMR and MCVL, which had decreasing levels with stage advancement. Their analysis was statistically significant for NLR, PLR, SIRI, SII, GLS, FAR, and MCVL, at the significance limit for LMR and IIC and beyond statistical significance for AISI and dNLR (Table 2).

Among previously known markers, Tukey’s post hoc multiple comparison test showed that the greatest differences between TNM stages I and IV were found for CAR (4.3 fold, mean diff. −16.89, *p* = 0.014), AISI (2.73 fold, mean diff. −932.02, *p* = 0.056), and SII (1.83 fold, mean diff. −872.09, *p* = 0.072). The smallest differences between stages I and IV were found for dNLR (1.16 fold), NLR (1.26 fold), and LMR (1.3 fold).

Regarding the TNM stage differences, for most of the analyzed inflammatory markers, the biggest differences were between stages III and IV, suggesting that once distant metastases appear, the level of inflammation increases (Table 2). According to Tukey’s post hoc test, the differences found for CAR (3.09 fold, mean diff. −14.82, *p* = 0.028) and SIRI (1.47 fold, mean diff. −223.84, *p* = 0.042) were statistically significant, which, together with AISI (1.54 fold, mean diff. −531.33, *p* = 0.073), had the biggest differences between stages III and IV. The smallest differences were found for dNLR (1.06 fold), NLR (1.1 fold), and LMR (1.12 fold), with almost equal levels between stages III and IV. Between the early stages, I and II, the biggest differences were found for AISI (1.40 fold, mean diff. −230.98, *p* = 0.087) and CAR (1.15 fold, mean diff. 0.75, *p* = 0.098); between stages II and III, the most significant differences were found for CAR (1.22 fold, mean diff. −1.32, *p* = 0.088) and AISI (1.21 fold, mean diff. −169.71, *p* = 0.099). The smallest differences between stages I and II were found for dNLR (1.01 fold) and SIRI (1.02 fold), with almost equal levels between the stages; between stages II and III, the smallest differences were found for LMR (1.04 fold) and SIRI (1.06 fold).

Regarding the new inflammatory markers, for MCVL, there was a statistically significant difference between stages I and IV (mean diff. −21.73, *p* = 0.031), with the biggest difference between stages I and II (1.38 fold, mean diff. −19.52, *p*= 0.137), and with slight differences between stages II and III (1.03 fold) and stages III and IV (1.01 fold). For IIC, there were no statistically significant differences between stage I and stage IV in the colorectal cancer patients (mean diff. −1.39, *p* = 0.056), with the biggest difference between stages III and IV (1.12 fold, mean diff. −0.56, *p* = 0.279) followed by a slightly smaller difference between stages II and III (1.11 fold, mean diff. −0.52, *p* = 0.184), and with the slightest difference between stage I and stage II (1.06 fold).

Regarding primary tumor extent (T-stage), the differences were statistically significant for PLR, SII, AISI, MCVL, GLS, and FAR, at the limit of statistical significance for IIC and with no statistical significance for LMR, SIRI, NLR, or NLR (Table 3).

Except for LMR, MCVL, and AISI, all markers had increasing levels the more invasive and advanced the tumors were. For AISI, there was a small decrease in T2 tumors compared to T1 tumors, followed by an increase in levels in T3 and T4 tumors. LMR and MCVL had decreasing levels in the more advanced tumors.

Among the known markers, after Tukey’s post hoc multiple comparison test, the biggest differences that were statistically significant between T1 and T2 tumors were found for CAR (2.54 fold, mean diff. 7.29, *p* = 0.023); those between T2 and T3 tumors were found for AISI (1.61 fold, mean diff. −357, *p* = 0.050); and those between T3 and T4 were found for CAR (1.76×, mean diff. −10.4, *p* = 0.035) and SII (1.72×, mean diff. −928.01, *p* = 0.044). The biggest differences that were not statistically significant between T1 and T2 were found for PLR (1.21 fold, mean diff. 19.91, *p* = 0.169), and those between T2 and T3 were found for CAR (1.34×, mean diff. −2.66, *p* = 0.066). The smallest differences between T1 and T2 were found for NLR (1.002 fold) and dNLR (1.09 fold); those between T2 and T3 were found for PLR (1.02 fold) and SIRI (1.07 fold); and those between T3 and T4 were found for LMR (1.05 fold) and NLR (1.08 fold).

Regarding the new inflammatory markers, the differences were not statistically significant. IIC had the biggest difference between T3 and T4 tumors (1.15 fold, mean diff. −0.91, *p* = 0.268), with the smallest being between T1 and T2 tumors (1.07×, mean diff. −0.33, *p* = 0.497). MCVL had almost equal differences between T1 and T2 tumors (1.15 fold, mean diff. 9.83, *p* = 0.370) and between T2 and T3 tumors (1.18×, mean diff. −9.80, *p* = 0.266), with a smaller difference between T3 and T4 tumors (1.08×, mean diff. −4.33, *p* = 0.591).

Regarding lymph node metastasis, all markers except LMR and MCVL had increasing levels in patients with more lymph nodes invaded. LMR and MCVL had decreasing levels in patients with more lymph nodes invaded. The statistical analysis showed that the SII, AISI, IIC, GLS, and FAR differences were statistically significant; MCVL was at the limit of statistical significance; and the NRL, PLR, LMR, SIRI, and dNLR differences were without statistical significance (Table 4).

Among the known markers, according to Tukey’s post hoc multiple comparison test, the biggest differences between N0 and N1 levels that were statistically significant were found for CAR (1.8 fold, mean diff. −6.02, *p* = 0.036); for AISI, the difference was not statistically significant (1.26 fold, mean diff. −227.06, *p* = 0.265). Between N1 and N2, the biggest differences were found for CAR (1.17 fold, mean diff. = −1.60, *p* = 0.160) and PLR (1.13 fold, mean diff. −26.89, *p* = 0.440) but without statistical significance.

Regarding the new inflammatory markers, the differences were not statistically significant. For IIC, the biggest difference was between N1 and N2 (1.19 fold, mean diff. −0.063, *p* = 0.260), with a small difference between N0 and N1 (1.04 fold mean diff. −0.29, *p* = 0.690). For MCVL, the differences were small between the N stages, with a slight difference between N0 and N1 (1.09 fold, mean diff. −6.64, *p* = 0.280) compared to N1 and N2 (1.07 fold, mean diff. −3.41, *p* = 0.320).

The differences in SII, AISI, CAR, and FAR were statistically significant regarding distant organ metastasis (Table 5). The biggest difference between markers, after the Independent *t*-test, was found for CAR (3.75 fold, mean diff. = −16.03, *p* = 0.002), followed by AISI (1.79 fold, mean diff. = −664.87, *p* = 0.018) and SII (1.53 fold, mean diff. = −664.16, *p* = 0.039). For SIRI, the differences were at the limit of statistical significance, with no statistical significance for NLR, PLR, LMR, and dNLR. In the case of the new markers, the differences were at the limit of statistical significance for MCVL (1.08 fold, mean diff. −4.43, *p* = 0.053) and without significance for IIC (1.21 fold, mean diff. −1.08, *p* = 0.089).

#### 3.1.2. Correlation of Inflammatory Markers with Pathological Tumor Differentiation Grade (G)

According to the pathological differentiation degree, the mean levels of analyzed markers PLR, SII, SIRI, AISI, IIC, CAR, and FAR increased as the tumor was less differentiated. The markers NLR, dNLR, LMR, and MCVL had decreased levels as the tumor was less differentiated, with statistical significance differences for PLR, CAR, and FAR; at the statistical limit for MCVL; and without statistical significance for NLR, LMR, SII, SIRI, AISI, dNLR, and IIC (Table 6).

Tukey’s post hoc multiple comparison test shows that no differences were statistically significant. The biggest differences between G1 and G2 were found for CAR (1.28 fold, mean diff. 1.96, *p* = 0.076) and FAR (1.10 fold, mean diff. 11.88, *p* = 0.710). Between G2 and G3, the biggest differences were also found for CAR (1.44 fold, mean diff, 0.056) and FAR (1.16 fold, mean diff. −11.59, *p* = 0.290). The smallest differences between G1 and G2 were found for PLR (1.01 fold) and SIRI (1.01 fold), and between G2 and G3, the smallest differences were found for SII (1.03 fold) and AISI (1.04 fold).

The new inflammatory markers had increasing levels as the tumor was less- differentiated, but with small differences and without statistical significance: for IIC, the difference between G1 and G2 was 1.05 fold (mean diff. 0.25, *p* = 0.79) and between G2 and G3 was 1.03 fold (mean diff. 0.16, *p* = 0.86); for MCVL, the differences between G1 and G2 were 1.09 fold (mean diff. −4.98, *p* = 0.61) and between G2 and G3, 1.05 fold (mean diff. −2.82, *p* = 0.87).

#### 3.1.3. Correlation of Inflammatory Markers with Clinicopathological Characteristics of Colorectal Patients

The clinicopathological characteristics of the colorectal cancer patients were analyzed (age, gender, and primary tumor location), and regarding these characteristics, their inflammatory markers levels were compared (Table 7).

Regarding patients’ age, NLR, PLR, SII, SIRI, AISI, dNLR, GLS, and FAR, the new IIC marker had higher levels in patients equal to or over the mean age of 70. In comparison, LMR and MCVL had higher levels in patients with a mean age under 70 years, without essential differences.

The largest differences among inflammatory markers, according to Independent *t*-test, were found for SIRI (1.15 fold, mean diff. 0.39, *p* = 0.25), which was statistically significant, and AISI (1.12 fold, mean diff. 113.01, *p* = 0.092), without statistical significance; the smallest difference was found for FAR (1.02 fold) and PLR (1.03 fold).

Regarding the new inflammatory markers, the differences were not statistically significant: IIC had the biggest difference between the age categories among all analyzed markers (1.16 fold, mean diff. −0.81, *p* = 0.19), and MCVL had a very small difference (1.05 fold, mean diff. −3.03, *p* = 0.49).

Regarding patient gender, NLR, LMR, SIRI, dNLR, CAR, and MCVL had higher levels in men compared to women, while PLR, SII, AISI, FAR, and IIC had higher levels in women.

The only inflammatory markers with a statistically significant difference and also with the biggest differences between genders were PLR (1.13 fold, mean diff. −25.09, *p* = 0.048) and FAR (1.12 fold, mean diff. −13.62, *p* = 0.018); the smallest differences were found for NLR and SIRI (1.01 fold). Regarding the new markers, the differences were very small, with no statistical significance: IIC with 1.03 fold (mean diff. = −0.18, *p* = 0.884) and MCVL with 1.05 fold (mean diff. = −0.15, *p* = 0.669).

Colorectal cancer patients were divided into two categories depending on the location of the primary tumor: patients with left colon tumors and patients with right colon tumors. Among the inflammatory markers, LMR and CAR had higher levels in patients with left colon tumors; the rest of the analyzed inflammatory markers had higher levels in patients with right colon tumors. The differences were not statistically significant. The biggest differences were found for AISI (1.23 fold mean diff. 216.75, *p* = 0.062) and SII (1.17 fold mean diff. −223.89, *p* = 0.058); the slightest difference was found for FAR and CAR (1.02 fold).

IIC had higher levels in patients with right colon tumors (1.05 fold mean diff. −0.31, *p* = 0.498), while MCVL had higher levels in patients with left colon tumors (1.10×, mean diff. −3.98, *p* = 0.196).

### 3.2. Diagnostic and Prognostic Value of Analyzed Inflammatory Markers in Colorectal Cancer Patients

**Figure 2:**
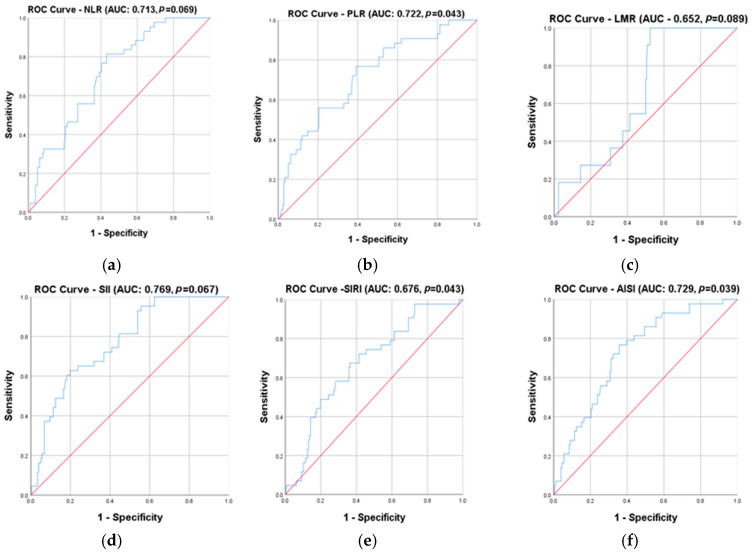

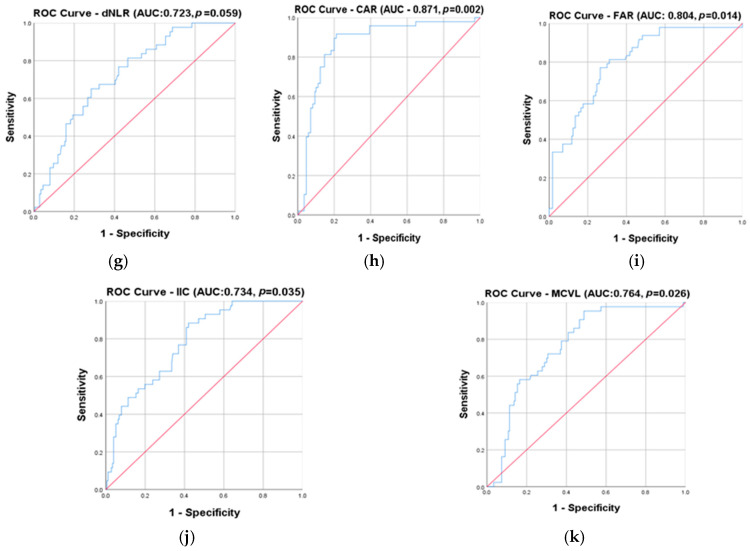
ROC (receiver operating characteristic) curve according to early (TNM I and II stages) and advanced (TNM III and IV stages) disease for (**a**) NLR; (**b**) PLR; (**c**) LMR; (**d**) SII; (**e**) SIRI; (**f**) AISI; (**g**) dNLR; (**h**) CAR; (**i**) FAR; (**j**) IIC; (**k**) MCVL.

Colorectal cancer patients were divided into two categories: patients with early disease (TNM I and II) and patients with advanced disease (TNM III and IV). ROC curves (Figure 2) were created for each analyzed marker, including the new markers, IIC and MCVL, to see their diagnostic capacity, sensitivity, and specificity in detecting advanced disease. The greatest area under the ROC curve (AUC) with statistical significance was found for CAR (87.1%, *p* = 0.002), followed by FAR (80.4%, *p* = 0.014). The smallest AUC with statistical significance was found for SIRI (67.6%, *p* = 0.043), and that without statistical significance was found for LMR (65.2%, *p* = 0.089) and NLR (71.1%, *p* = 0.069).

Regarding the newly analyzed inflammatory markers, both were statistically significant: MCVL had an AUC of 76.4% (*p* = 0.026), and IIC had an AUC of 73.4% (*p* = 0.035).

For each of the analyzed inflammatory markers, cut-off levels were chosen according to the sensitivity and specificity shown by the ROC curves in the diagnosis of advanced forms of cancer. Among the known markers, CAR had the best sensitivity and specificity, with 91.7% sensitivity and 78.8% specificity (cut-off value 6.59), followed by FAR with 81.4% sensitivity and 68.8% specificity (119.5 cut-off value) and dNLR with 80.8% sensitivity and 56.4 specificity % (cut-off value 2.42). The other inflammatory markers in our study: NLR had a sensitivity of 76.7% and a specificity of 56.8% (4.14 cut-off value), PLR had a sensitivity of 78.1% and a specificity of 60.8% (198.36 cut-off value), LMR had a sensitivity of 72.7% and a specificity of 50.5% (3.31 cut-off value), SII had a sensitivity of 78.4% and a specificity of 56.7% (cut-off value 1312.14), SIRI had a sensitivity of 74.4% and a specificity of 55.4% (cut-off value 2.41), and AISI had a sensitivity of 79.8% and a specificity of 58.7% (cut-off values of 906.21).

Among the newly analyzed inflammatory markers, MCVL had a sensitivity of 83.7% and a specificity of 60% (cut-off values of 50.49), and IIC had a sensitivity of 79.1% and a specificity of 61.9% (cut-off values of 5.45).

### 3.3. Comparison Between Inflammatory Markers Regarding Mean Survival Time in Colorectal Cancer Patients

**Figure 3:**
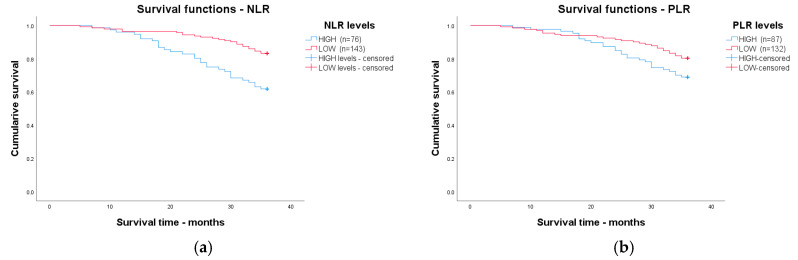

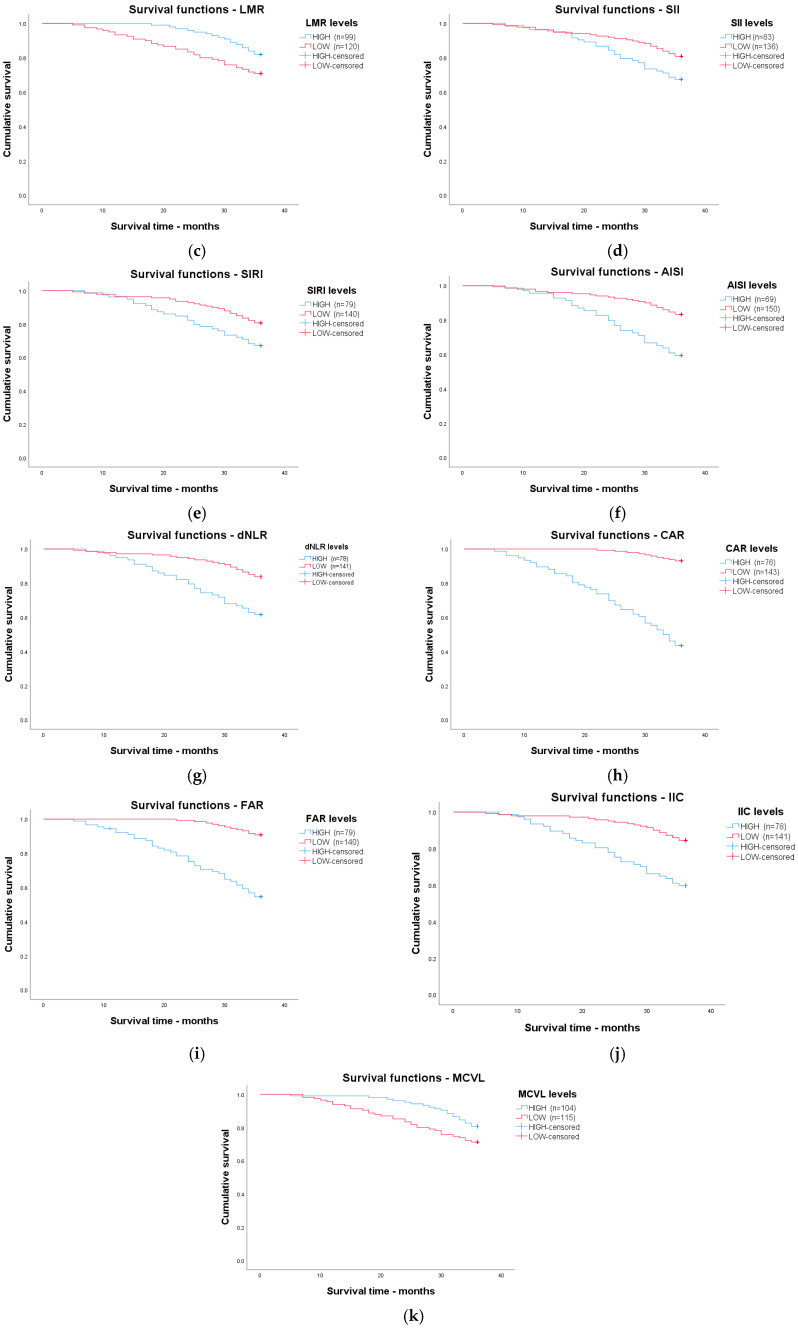
Kaplan–Meier curve survival analysis regarding high and low levels of (**a**) NLR; (**b**) PLR; (**c**) LMR; (**d**) SII; (**e**) SIRI; (**f**) AISI; (**g**) dNLR; (**h**) CAR; (**i**) FAR; (**j**) IIC; (**k**) MCVL.

The patients were followed for 36 months and divided depending on each analyzed marker’s high or low levels. Figure 3 shows the Kaplan–Meier analysis with a log-rank test of analyzed markers. The lowest survival time was for patients with high levels of CAR, with a mean survival time of 26.4 months, followed by those with high levels of FAR, with a mean survival time of 27.6 months, and high levels of AISI, with a mean survival time of 29.6 months. The longest survival time was for patients with low levels of CAR, with a mean survival time of 35.5 months, followed by patients with low levels of FAR, with a mean survival time of 35.1 months, and patients with low levels of dNLR, with a mean survival time of 34.1 months.

Of the newly analyzed markers for patients with high levels of IIC, the mean survival time was 29.5 months, and for patients with low levels of IIC, the mean survival time was 34.1 months. The mean survival time for MCVL patients with high levels was 34.3 months; for patients with low levels, the mean survival time was 30.8 months.

Table 8 shows the 36-month survival time for all analyzed markers, depending on their high or low levels. The differences for all analyzed markers were statistically significant, except for PLR, which was at the statistical significance limit.

## 4. Discussion

From all analyzed markers, CAR correlates the most with TNM stages, pathological differentiation degree, and mean survival time in colorectal cancer patients. It also had the highest diagnostic capacity, sensitivity, specificity, and survival time of all analyzed inflammatory markers. It is followed by FAR, which had a diagnostic capacity close to that of CAR, and also correlated with TNM stages, pathological differentiation degree, and mean survival time. This means that the levels of inflammatory circulating proteins are sensitive to the changes that occur in colorectal cancer.

All hematological markers that are composed of complete blood count cells correlate with the TNM stage, with increasing levels in more advanced stages (except LMR, which had decreasing levels in more advanced stages). Except for AISI, all markers were associated with the primary tumor extent (T stage), with increasing levels in more invasive tumors. All inflammatory markers correlate with lymph node metastases, with increasing levels as more lymph nodes were affected (except LMR with decreasing levels). Also, all the analyzed markers correlate with distant organ metastases, with increasing levels (except LMR, which had decreased levels), with a significant increase found in some of the markers in patients with distant metastases. This might suggest that changes in blood cell levels and the modified balance between innate and adaptive immunity play an important role in the inflammation that occurs in the evolution and characteristics of colorectal cancer.

Regarding the newly analyzed hematological markers, the IIC and MCVL levels correlate with the TNM stage; with the extent of the primary tumor (T), lymph node metastasis (N), and distant metastases (M); and with the pathological differentiation degree (G). Also, the analysis of the ROC curves showed that these markers have an important diagnostic significance in differentiating the advanced stages from the early ones, with a sensitivity of 76% in the case of MCVL and 73% in the case of IIC, and can be used as prognostic markers. This means that in addition to the inflammatory cells’ role, another important factor in colorectal cancer may be represented by the changes that occur at the erythrocyte level in response to the pro-inflammatory state or the blood loss from the tumor level in colorectal cancer.

Neutrophils represent the main cells in innate immune response, playing an important role in phagocytosis and cytokine secretion [13]. In cancers, neutrophils have a strong pro-tumor role in suppressing B and T lymphocytes; producing cytokines; and stimulating tumor cell growth, proliferation, and metastasis [42,43,44,45]. Monocytes, especially through tumor-associated macrophages (TMAs), produce cytokines and growth factors at the tumor level, having a role in the anti-immune response stimulating angiogenesis, tumor cell migration, and metastasis [46,47,48,49,50]. Lymphocytes represent acquired immunity and have an anti-tumor role in stimulating the immune system and inducing apoptosis in tumor cells by inhibiting the production of cytokines, with low levels being associated with worse cancer pre-treatment prognosis [51,52,53,54,55]. Platelets interact with neutrophils and monocytes by secreting growth factors, with a role in proliferation, cell migration, and especially metastasis, by helping tumor cells reach the site of metastasis [56,57,58,59,60,61]. Increased platelet numbers can also have a negative prognosis by association with the procoagulant status and the formation of thrombosis in different types of cancer [62].

The neutrophil-to-lymphocyte ratio is a marker that has been associated with the presence of lymph node metastases and distant metastases and is associated with an unfavorable prognosis and a poor treatment response in the case of colorectal, esophageal, pulmonary, renal, and pancreatic cancer [11,63,64]. The lymphocyte-to-monocyte ratio is an inflammation marker that has been increasingly used in various chronic diseases, such as atheromatous disease and ischemic stroke, and in different types of cancers such as squamous esophageal, lung, pancreatic, and colorectal cancer [3,27,48,50,53]. The platelet-to-lymphocyte ratio is a marker that has been studied in various types of cancer, such as digestive (esophageal, colorectal, and pancreatic cancers), lung, breast prostate, ovary, and cervical cancers, and increased levels are associated with poor prognosis, low disease-free survival, and advanced disease—lymph node and distant metastases [48,65,66,67,68,69,70]. Increased levels of NLR, PLR, and LMR are associated with a negative prognosis in multiple cancers, including colorectal cancer [11,53,66,68].

The other markers, the systemic immune inflammation index, the systemic inflammatory response index, the aggregate index of systemic inflammation, and the derived neutrophil-to-lymphocyte ratio, are determined by ratios between more than two types of complete blood count cells, including neutrophils, lymphocytes, monocytes, and platelets. They can more clearly show the inflammation status linked with various inflammatory diseases and cancers [29,33,71,72,73]. Their levels correlate with advanced forms of colorectal cancer and with other types of cancer, such as bladder cancer, lung cancer, and prostate cancer [30,31,32,72,73,74,75,76,77,78,79]

In this study, we demonstrated that these hematological markers correlate with TNM stage and pathological differentiation degree in patients with colorectal cancer and could represent useful markers in diagnostics and prognostics in colorectal cancer patients.

Red cell distribution width (RDW) measures the variation in erythrocyte size and is used mainly in diagnosing anemia or other blood disorders [80,81]. RDW has increased levels in various diseases, including hematological diseases, inflammatory diseases, and cancers, including colorectal cancer, and can be used as a prognostic factor [80,82,83,84]. Mean corpuscular volume (MCV) measures the average size of red blood cells and is an important factor in diagnosing anemia, helping in classifying anemia. MCV is often modified in cancers, most of the time with low levels, but can also have high levels, mainly after oncological treatment; very low or very high levels of MCV are associated with poor prognosis in cancers [85,86,87].

Two new hematological inflammatory markers, the cumulative inflammatory index (IIC) and the ratio between the mean corpuscular volume and lymphocytes (MCVL), were developed. They reflect the changes between erythrocyte characteristics (RDW and MCV levels) and leucocytes (neutrophils and lymphocytes). These markers have been used in the prognosis of inflammatory diseases such as acute pancreatitis and ulcerative colitis [34,35]. This study demonstrated that both new inflammatory markers can be used for diagnostic and prognostic purposes, correlating with the TNM stages and the pathological differentiation degree in colorectal cancer patients. But, although IIC had increasing levels with an advancement in TNM stage and also in less differentiated tumors, MCVL had decreasing levels with an advancement in TNM stage and less differentiated tumors, showing that although there are inflammatory changes in colorectal cancer, there is also blood loss through bleeding at the tumor level, lowering the MCV level.

Fibrinogen and C-reactive protein are circulating proteins with increased levels in inflammatory states and cancers [21,22,88,89,90,91]. Albumin is a plasmatic protein with low levels in inflammatory states and cancers [19,92,93]. These proteins are synthesized by hepatocytes, and their levels change with inflammation, especially under the influence of cytokines, which increase the level of CRP and fibrinogen and decrease the level of albumin [94,95,96]. Fibrinogen, an acute-phase protein, is present at the tumor level and seems to have an important role in carcinogenesis in processes like angiogenesis, metastasis promotion, and thrombosis [24,97,98,99]. C-reactive protein, another plasmatic inflammatory protein, has high levels in cancer and promotes metastasis and suppression of the immune response [100,101]. Albumin indicates patients’ nutritional status and has an anti-inflammatory and antioxidant role [23,102,103]. Hypoalbuminemia occurs in cancer, with its primary mechanism being inhibiting its synthesis through pro-inflammatory cytokines [40,104]. FAR and CAR had increased levels in various cancers, including colorectal cancer, and are related to poor prognosis, reinforcing the idea of inflammation’s role in cancers [19,24,40,95,105,106,107,108,109].

The inflammatory markers analyzed in this study are easy to determine right from admission in patients suspected of colorectal cancer. These markers are based on standard blood tests and have the advantage that they are easy to repeat throughout the patients’ disease evolution.

In this study, we showed that these inflammatory markers could help in the diagnosis and prognosis of colorectal cancer patients, and by comparing them, we showed which one correlates better with colorectal cancer, which one has better sensitivity and specificity in the diagnosis of advanced forms of cancer, and also which one better predicts survival time. This is useful in choosing which marker to use when we have a newly diagnosed patient with colorectal cancer.

Also, in this study, we tested if the levels of the new markers IIC and MCVL correlate with colorectal cancer and if they can help the diagnosis and prognosis of patients with this type of cancer, with positive results. These markers correlate with the disease stage and with the tumor characteristics, having relatively significant sensitivity levels in the diagnosis of advanced forms of colorectal cancer. Also, they correlate with survival time with important differences between those with high levels and low levels.

This study had several shortcomings: the group of patients was not heterogeneous, with patients from only one Romanian region included in this study; this study was also not multicentric; the number of patients included in this study was not very big; and the patients were not followed up for the periodic blood tests to see the evolution of these markers simultaneously with the evolution of the disease. So, new studies that can cover all these shortcomings must be performed to better analyze these inflammatory markers in colorectal cancer.

Although the diagnostic and prognostic role of most of the analyzed markers in this study in colorectal cancer was studied before, as far as we know, this is the first study in which the levels of all of them are compared in colorectal cancer patients. Also, it is the first study in which the new IIC and MCVL markers are analyzed in patients with this type of cancer.

## 5. Conclusions

In this study, we have underlined the important role of inflammation in colorectal cancer, analyzing markers based on the changes in the levels of inflammatory cells that are components of complete blood count, with simple (NLR, LMR, and PLR) or more complex (SII, SIRI, AISI, and dNLR) ratios between these cells. Furthermore, we have analyzed new hematological markers (IIC and MCVL) and markers based on changes in inflammatory circulating proteins (CAR and FAR). All these inflammatory markers correlate with disease stage and the clinicopathological and tumor characteristics in colorectal cancer patients. Also, we demonstrated the diagnostic and prognostic role of these markers in colorectal cancer patients, showing which one correlates better and has greater sensitivity and specificity in the diagnosis of advanced forms and in predicting survival time. This can help to guide their use for better diagnosis and a more accurate prognosis in patients with this type of cancer.

## Figures and Tables

**Table 1:** Case distribution according to colorectal adenocarcinoma patients’ characteristics.

Patient Characteristics		No. of Cases	Total
Age	≥70 Years old	128 (58.4%)	219 (100%)
	<70 years old	91 (41.5%)	
Gender	Male	127 (57.9%)	219 (100%)
	Female	92 (42%)	
Tumor Localization	Rectum	50 (22.8%)	219 (100%)
	Sigmoid	71 (32.4%)	
	Descending	18 (8.21%)	
	Transverse	21 (9.5%)	
	Ascending	49 (22.3%)	
	Cecum	10 (4.5%)	
TNM Classification	I	32 (14.6%)	219 (100%)
	II	80 (36.5%)	
	III	59 (26.9%)	
	IV	48 (21.9%)	
T Stage	T1	12 (5.4%)	219 (100%)
	T2	21 (9.5%)	
	T3	143 (65.2%)	
	T4	43 (19.6%)	
N Stage	N0	122 (55.7%)	219 (100%)
	N1	64 (29.2%)	
	N2	33 (15%)	
M Stage	M0	171 (78%)	219 (100%)
	M1	48 (21.9%)	
Tumor Pathological Grade	G1	42 (19.1%)	219 (100%)
	G2	125 (57%)	
	G3	52 (23.7%)	

**Table 2:** Mean levels of inflammation markers according to TNM stages.

TNM Stage	I	II	III	IV	*p*-Value
No. of Patients	32	80	59	48	
Marker					
NLR	3.73 (±1.79)	3.88 (±2.86)	4.3 (±2.5)	4.73 (±2.12)	0.023 *
PLR	182.74 (±82.09)	184.49 (±94.51)	205.34 (±131.8)	237.6 (±113.22)	0.017 *
LMR	3.76 (±2.71)	3.38 (±1.33)	3.25 (±1.57)	2.88 (±1.45)	0.048 *
SII	1041.1 (±901.67)	1204.74 (±773.89)	1364.86 (±1249.61)	1913.19 (±1241.55)	0.041 *
SIRI	2.3 (±2.09)	2.36 (±1.58)	2.52 (±3.37)	3.72 (±2.81)	0.036 *
AISI	571.49 (±490.74)	802.47 (±707.6)	972.18 (±923.89)	1503.51 (±1217.65)	0.060 *
dNLR	2.5 (±1.38)	2.53 (±1.13)	2.74 (±1.68)	2.92 (±1.03)	0.077 *
IIC	4.73 (±2.29)	5.03 (±4.54)	5.61 (±3.39)	6.32 (±3.32)	0.046 *
MCVL	70.72 (±33.03)	51.2 (±20.13)	49.66 (±15.12)	48.99 (±22.65)	0.036 *
CAR	5.0 (±2.68)	5.75 (±4.82)	7.07 (±6.5)	21.89 (±18.85)	<0.001 *
FAR	97.1 (±36.63)	103.1 (±24.79)	121.15 (±31.22)	145.4 (±38.28)	0.003 *

* One-way ANOVA.

**Table 3:** Average levels of inflammation markers according to primary tumor extent—T stage.

Tumor Invasion	T1	T2	T3	T4	*p*-Value
No. of Patients	12	21	143	43	
Marker					
NLR	4.22 (±2.55)	4.23 (±1.73)	4.74 (±2.34)	5.12 (±2.33)	0.067 *
PLR	165.48 (±73.18)	187.21 (±102.52)	192.87 (±103.66)	272.31 (±129.86)	0.033 *
LMR	3.99 (±1.57)	3.59 (±2.25)	2.94 (±1.52)	2.8 (±1.22)	0.250 *
SII	982.46 (±691.25)	1113.24 (±1092.64)	1250.75 (±1172.48)	2152.78 (±1452.35)	0.029 *
SIRI	2.07 (±1.53)	2.37 (±1.82)	2.56 (±1.8)	3.56 (±2.74)	0.810 *
AISI	566.85 (±474.38)	556.0 (±367.61)	913.01 (±459.32)	1544.59 (±1055.29)	0.022 *
dNLR	2.43 (±1.15)	2.65 (±0.995)	2.81 (±2.51)	3.26 (±1.28)	0.063 *
IIC	4.82 (±3.4)	5.16 (±4.77)	5.7 (±2.4)	6.61 (±4.23)	0.056 *
MCVL	73.16 (±34.43)	63.33 (±31.21)	53.53 (±20.58)	49.2 (±19.5)	0.041 *
CAR	2.99 (±1.89)	7.62 (±4.73)	10.28 (±9.06)	18.1 (±10.29)	<0.001 *
FAR	93.1 (±45.91)	100.35 (±30.34)	114.83 (±34.97)	136.03 (±30.71)	0.015 *

* One-way ANOVA.

**Table 4:** Average levels of inflammation markers according to lymph node metastasis—N stage.

Lymph Node Metastasis	N0	N1	N2	*p*-Value
No. of Patients	122	64	33	
Marker				
NLR	3.99 (±2.67)	4.12 (±2.0)	4.4 (±2.17)	0.182 *
PLR	198.19 (±118.61)	200.52 (±91.68)	227.41 (±122.6)	0.226 *
LMR	3.56 (±2.38)	3.29 (±1.47)	2.97 (±1.29)	0.483 *
SII	1275.8 (±1058.29)	1485.43 (±963.45)	1657.65 (±1310.36)	0.004 *
SIRI	2.49 (±1.98)	2.95 (±2.31)	3.04 (±2.43)	0.080 *
AISI	872.33 (±623.72)	1099.39 (±999.32)	1175.45 (±1128.72)	0.004 *
dNLR	2.57 (±1.42)	2.64 (±0.999)	2.87 (±1.34)	0.157 *
IIC	4.96 (±2.48)	5.19 (±4.12)	6.2 (±3.5)	0.043 *
MCVL	56.17 (±25.06)	51.53 (±25.21)	48.12 (±17.34)	0.058 *
CAR	6.62 (±6.27)	12.14 (±7.03)	14.24 (±13.82)	<0.001 *
FAR	105.52 (±33.45)	127.3 (±35.17)	135.28 (±33.83)	<0.001 *

* One-way ANOVA.

**Table 5:** Average levels of inflammation markers according to distant organ metastasis—M stage.

Distant Metastasis	M0	M1	*p*-Value
No. of Patients	171	48	
Marker			
NLR	3.91 (±2.47)	4.73 (±2.12)	0.840 *
PLR	193.64 (±110.1)	237.6 (±113.22)	0.097 *
LMR	3.53 (±2.12)	2.88 (±1.45)	0.062 *
SII	1249.03 (±1218.04)	1913.19 (±1241.55)	0.039 *
SIRI	2.42 (±1.63)	3.72 (±2.81)	0.050 *
AISI	838.64 (±414.27)	1503.51 (±1217.65)	0.018 *
dNLR	2.55 (±1.36)	2.92 (±1.03)	0.719 *
IIC	5.04 (±3.68)	6.12 (±3.32)	0.089 *
MCVL	54.09 (±25.04)	49.66 (±15.12)	0.053 *
CAR	5.86 (±4.17)	21.89 (±18.85)	0.002 *
FAR	108.24 (±30.96)	145.4 (±38.28)	0.027 *

* Independent *t*-test.

**Table 6:** Average levels of inflammation markers according to pathological tumor differentiation grade (G).

Pathological Differentiation Grade	G1	G2	G3	*p*-Value
No. of Patients	42	125	52	
Marker				
NLR	4.23 (±3.07)	4.13 (±2.41)	3.86 (±1.76)	0.469 *
PLR	183.15 (±89.11)	186.18 (±100.18)	217.15 (±121.88)	0.15 *
LMR	3.63 (±1.77)	3.41 (±2.31)	3.14 (±1.3)	0.363 *
CAR	6.87 (±5.13)	8.83 (±4.34)	12.72 (±9.5)	0.007 *
FAR	104.01 (±39.6)	115.89 (±34.66)	127.48 (±33.75)	<0.001 *
SII	1302.63 (±1096.02)	1389.87 (±1193.15)	1434.45 (±1334.79)	0.907 *
SIRI	2.66 (±1.95)	2.69 (±2.28)	2.85 (±2.5)	0.617 *
AISI	912.31 (±853.6)	972.95 (±864.01)	1013.32 (±951.82)	0.664 *
dNLR	2.77 (±1.95)	2.65 (±1.17)	2.49 (±0.887)	0.372 *
IIC	4.99 (±2.92)	5.24 (±3.73)	5.4 (±3.87)	0.467 *
MCVL	55.93 (±24.18)	50.95 (±25.27)	48.13 (±18.25)	0.054 *

* One-way ANOVA.

**Table 7:** Average levels of inflammation markers according to age, gender, and primary tumor location.

	Age			Gender			Primary Tumor Location		
Marker	≥70 Years Old 128 Patients	<70 Years Old 91 Patients	*p*-Value	Men 127 Patients	Woman 92 Patients	*p*-Value	Left Colon 138 Patients	Right Colon 80 Patients	*p*-Value
NLR	4.29 (±2.77)	3.8 (±1.77)	0.172 *	4.12 (±2.32)	4.04 (±2.55)	0.843 *	3.99 (±2.26)	4.28 (±2.66)	0.172 *
PLR	205.89 (±107.67)	199.59 (±118.38)	0.128 *	192.74 (±105.15)	217.81 (±119.94)	0.048 *	193.62 (±102.2)	220.94 (±126.32)	0.128 *
LMR	3.3 (±2.2)	3.52 (±1.71)	0.222 *	3.63 (±2.51)	3.32 (±1.54)	0.365 *	3.51 (±2.21)	3.17 (±1.61)	0.222 *
SII	1457.9 (±1440.07)	1305.56 (±922.49)	0.08 *	1365.26 (±1185.67)	1435.1 (±1341.65)	0.529 *	1316.88 (±1138.06)	1540.77 (±1424.36)	0.058 *
SIRI	2.87 (±1.17)	2.48 (±1.89)	0.254 *	2.72 (±2.08)	2.69 (±2.42)	0.177 *	2.58 (±2.28)	2.96 (±2.35)	0.254 *
AISI	1031.32 (±616.57)	918.32 (±820.4)	0.092 *	947.6 (±825.02)	1035.12 (±795.86)	0.698 *	908.38 (±779.14)	1125.13 (±827.19)	0.062 *
dNLR	2.73 (±1.51)	2.49 (±0.925)	0.06 *	2.66 (±1.35)	2.6 (±1.24)	0.845 *	2.57 (±1.31)	2.76 (±1.29)	0.06 *
IIC	5.61 (±4.06)	4.8 (±2.86)	0.196 *	5.38 (±4.03)	5.2 (±3.32)	0.884 *	5.18 (±3.47)	5.49 (±3.89)	0.498 *
MCVL	51.35 (±22.98)	54.38 (±23.49)	0.498 *	53.21 (±23.39)	53.06 (±23.25)	0.669 *	55.09 (±25.9)	49.77 (±17.7)	0.196 *
GLS	9.59 (±6.57)	9.12 (±6.88)	0.345 *	9.55 (±6.62)	9.18 (±6.81)	0.616 *	9.53 (±6.68)	9.27 (±6.81)	0.345 *
FAR	117.61 (±37.61)	114.75 (±33.97)	0.119 *	110.67 (±36.08)	124.29 (±34.76)	0.018 *	115.53 (±38.85)	118.64 (±30.48)	0.119 *

* Independent *t*-test.

**Table 8:** Mean survival time regarding high and low levels of inflammatory markers.

Marker	Survival Time		χ^2^	*p*-Value
	High Levels	Low Levels		
NLR	30 months (76 patients)	34 months (143 patients)	13.68	<0.001 *
PLR	31.3 months (87 patients)	33.6 months (132 patients)	3.8	0.051 *
LMR	34.7 months (99 patients)	30.7 months (120 patients)	4.41	0.036 *
SII	31 months (83 patients)	33.7 months (136 patients)	5.31	0.021 *
SIRI	30 months (79 patients)	33.7 months (140 patients)	5.59	0.017 *
AISI	29.1 months (69 patients)	34.03 months (150 patients)	16.1	<0.001 *
dNLR	29.6 months (78 patients)	34.2 months (141 patients)	15	<0.001 *
IIC	29.5 months (77 patients)	34.1 months (142 patients)	19.2	<0.001 *
MCVL	34.3 months (104 patients)	30.8 months (115 patients)	4.37	<0.001 *
CAR	26.4 months (76 patients)	35.5 months (143 patients)	78.5	<0.001 *
FAR	27.6 months (79 patients)	35.1 months (140 patients)	42.64	<0.001 *

* Log-rank test.

## Data Availability

The data are contained within this article.

## Abbreviations

The following abbreviations are used in this manuscript:

NLR	Neutrophil-to-lymphocyte ratio
PLR	Platelet-to-lymphocyte ratio
LMR	Lymphocyte-to-monocyte ratio
SII	Systemic immune inflammation index
SIRI	Systemic inflammatory response index
AISI	Aggregate index of systemic inflammation
dNLR	Derived neutrophil-to-lymphocyte ratio
IIC	Cumulative inflammatory index
MCVL	Mean corpuscular volume and lymphocytes
TNM	Tumor, node, metastasis

## References

[1] Kuipers E.J., Grady W.M., Lieberman D., Seufferlein T., Sung J.J., Boelens P.G., van de Velde C.J.H., Watanabe T. (2015). Colorectal cancer. Nat. Rev. Dis. Primers.

[2] Hislop G. (2000). Trends and risk factors for colorectal cancer. Br. Columbia Med. J..

[3] Weitz J., Koch M., Debus J., Höhler T., Galle P.R., Büchler M.W. (2005). Colorectal cancer. Lancet.

[4] Beaugerie L., Itzkowitz S.H. (2015). Cancers complicating inflammatory bowel disease. N. Engl. J. Med..

[5] Coussens L.M., Werb Z. (2002). Inflammation and cancer. Nature.

[6] Mantovani A., Allavena P., Sica A., Balkwill F. (2008). Cancer-related inflammation. Nature.

[7] Șerban R.E., Boldeanu M.V., Florescu D.N., Ionescu M., Șerbănescu M.S., Boldeanu L., Florescu M.M., Stepan M.D., Obleagă V.C., Constantin C. (2024). Comparison between Substance P and Calcitonin Gene-Related Peptide and Their Receptors in Colorectal Adenocarcinoma. J. Clin. Med..

[8] Pavel C., Diculescu M.M., Ilie M., Plotogea O.M., Sandru V., Enache V., Gheonea D.I., Jichitu A., Constantinescu A., Serban R.E. (2025). Immunohistochemistry Analysis in Inflammatory Bowel Disease-Should We Bring to Light Interleukin-10?. Biomedicines.

[9] Brennan C.A., Garrett W.S. (2016). Gut Microbiota, Inflammation, and Colorectal Cancer. Annu. Rev. Microbiol..

[10] Roxburgh C.S., McMillan D.C. (2010). Role of systemic inflammatory response in predicting survival in patients with primary operable cancer. Future Oncol..

[11] Vigano A., Bruera E., Jhangri G.S., Newman S.C., Fields A.L., Suarez-Almazor M.E. (2000). Clinical survival predictors in patients with advanced cancer. Arch. Intern. Med..

[12] Templeton A.J., McNamara M.G., Šeruga B., Vera-Badillo F.E., Aneja P., Ocaña A., Leibowitz-Amit R., Sonpavde G., Knox J.J., Tran B. (2014). Prognostic role of neutrophil-to-lymphocyte ratio in solid tumors: A systematic review and meta-analysis. J. Natl. Cancer Inst..

[13] Florescu D.N., Boldeanu M.V., Șerban R.E., Florescu L.M., Serbanescu M.S., Ionescu M., Streba L., Constantin C., Vere C.C. (2023). Correlation of the Pro-Inflammatory Cytokines IL-1β, IL-6, and TNF-α, Inflammatory Markers, and Tumor Markers with the Diagnosis and Prognosis of Colorectal Cancer. Life.

[14] Zahorec R. (2021). Neutrophil-to-lymphocyte ratio, past, present and future perspectives. Bratisl. Lek. Listy.

[15] Mishalian I., Bayuh R., Eruslanov E., Michaeli J., Levy L., Zolotarov L., Singhal S., Albelda S.M., Granot Z., Fridlender Z.G. (2014). Neutrophils recruit regulatory T-cells into tumors via secretion of CCL17—A new mechanism of impaired antitumor immunity. Int. J. Cancer.

[16] Khandia R., Munjal A. (2020). Interplay between inflammation and cancer. Adv. Protein Chem. Struct. Biol..

[17] Kim S.J., Davis R.P., Jenne C.N. (2018). Platelets as Modulators of Inflammation. Semin. Thromb. Hemost..

[18] Gasparyan A.Y., Ayvazyan L., Mukanova U., Yessirkepov M., Kitas G.D. (2019). The Platelet-to-Lymphocyte Ratio as an Inflammatory Marker in Rheumatic Diseases. Ann. Lab. Med..

[19] Wen Y., Yang J., Han X. (2021). Fibrinogen-to-Albumin Ratio is Associated with All-Cause Mortality in Cancer Patients. Int. J. Gen. Med..

[20] Tsilidis K.K., Branchini C., Guallar E., Helzlsouer K.J., Erlinger T.P., Platz E.A. (2008). C-reactive protein and colorectal cancer risk: A systematic review of prospective studies. Int. J. Cancer.

[21] Gabay C., Kushner I. (1999). Acute-phase proteins and other systemic responses to inflammation. N. Engl. J. Med..

[22] Lin Y., Liu Z., Qiu Y., Zhang J., Wu H., Liang R., Chen G., Qin G., Li Y., Zou D. (2018). Clinical significance of plasma D-dimer and fibrinogen in digestive cancer: A systematic review and meta-analysis. Eur. J. Surg. Oncol..

[23] Gupta D., Lis C.G. (2010). Pretreatment serum albumin as a predictor of cancer survival: A systematic review of the epidemiological literature. Nutr. J..

[24] Barone B., Napolitano L., Reccia P., De Luca L., Morra S., Turco C., Melchionna A., Caputo V.F., Cirillo L., Fusco G.M. (2022). Preoperative Fibrinogen-to-Albumin Ratio as Potential Predictor of Bladder Cancer: A Monocentric Retrospective Study. Medicina.

[25] Pfensig C., Dominik A., Borufka L., Hinz M., Stange J., Eggert M. (2016). A New Application for Albumin Dialysis in Extracorporeal Organ Support: Characterization of a Putative Interaction Between Human Albumin and Proinflammatory Cytokines IL-6 and TNFα. Artif. Organs.

[26] Tian C., Song W., Tian X., Sun Y. (2018). Prognostic significance of platelet-to-lymphocyte ratio in patients with ovarian cancer: A meta-analysis. Eur. J. Clin. Investig..

[27] Tham T., Olson C., Khaymovich J., Herman S.W., Costantino P.D. (2018). The lymphocyte-to-monocyte ratio as a prognostic indicator in head and neck cancer: A systematic review and meta-analysis. Eur. Arch. Otorhinolaryngol..

[28] Zhou Y., Wei Q., Fan J., Cheng S., Ding W., Hua Z. (2018). Prognostic role of the neutrophil-to-lymphocyte ratio in pancreatic cancer: A meta-analysis containing 8252 patients. Clin. Chim. Acta..

[29] Wu J., Yan L., Chai K. (2021). Systemic immune-inflammation index is associated with disease activity in patients with ankylosing spondylitis. J. Clin. Lab. Anal..

[30] Li C., Tian W., Zhao F., Li M., Ye Q., Wei Y., Li T., Xie K. (2018). Systemic immune-inflammation index, SII, for prognosis of elderly patients with newly diagnosed tumors. Oncotarget.

[31] Wei L., Xie H., Yan P. (2020). Prognostic value of the systemic inflammation response index in human malignancy: A meta-analysis. Medicine.

[32] Wang H.K., Wei Q., Yang Y.L., Lu T.Y., Yan Y., Wang F. (2023). Clinical usefulness of the lymphocyte-to-monocyte ratio and aggregate index of systemic inflammation in patients with esophageal cancer: A retrospective cohort study. Cancer Cell Int..

[33] Yang T., Hao L., Yang X., Luo C., Wang G., Cai C.L., Qi S., Li Z. (2021). Prognostic value of derived neutrophil-to-lymphocyte ratio (dNLR) in patients with non-small cell lung cancer receiving immune checkpoint inhibitors: A meta-analysis. BMJ Open.

[34] Poenariu I.S., Boldeanu L., Ungureanu B.S., Caragea D.C., Cristea O.M., Pădureanu V., Siloși I., Ungureanu A.M., Statie R.C., Ciobanu A.E. (2023). Interrelation of Hypoxia-Inducible Factor-1 Alpha (HIF-1 α) and the Ratio between the Mean Corpuscular Volume/Lymphocytes (MCVL) and the Cumulative Inflammatory Index (IIC) in Ulcerative Colitis. Biomedicines.

[35] Radulescu P.M., Davitoiu D.V., Baleanu V.D., Padureanu V., Ramboiu D.S., Surlin M.V., Bratiloveanu T.C., Georgescu E.F., Streba C.T., Mercut R. (2022). Has COVID-19 Modified the Weight of Known Systemic Inflammation Indexes and the New Ones (MCVL and IIC) in the Assessment as Predictive Factors of Complications and Mortality in Acute Pancreatitis?. Diagnostics.

[36] Ranzani O.T., Zampieri F.G., Forte D.N., Azevedo L.C., Park M. (2013). C-reactive protein/albumin ratio predicts 90-day mortality of septic patients. PLoS ONE.

[37] Kaplan M., Ates I., Akpinar M.Y., Yuksel M., Kuzu U.B., Kacar S., Coskun O., Kayacetin E. (2017). Predictive value of C-reactive protein/albumin ratio in acute pancreatitis. Hepatobiliary Pancreat. Dis. Int..

[38] Glapa-Nowak A., Szczepanik M., Banaszkiewicz A., Kwiecień J., Szaflarska-Popławska A., Grzybowska-Chlebowczyk U., Osiecki M., Kierkuś J., Dziekiewicz M., Walkowiak J. (2022). C-Reactive Protein/Albumin Ratio at Diagnosis of Pediatric Inflammatory Bowel Disease: A Retrospective Multi-Center Study. Med. Sci. Monit..

[39] Tominaga T., Nonaka T., Sumida Y., Hidaka S., Sawai T., Nagayasu T. (2016). The C-Reactive Protein to Albumin Ratio as a Predictor of Severe Side Effects of Adjuvant Chemotherapy in Stage III Colorectal Cancer Patients. PLoS ONE.

[40] Fang L., Yan F.H., Liu C., Chen J., Wang D., Zhang C.H., Lou C.J., Lian J., Yao Y., Wang B.J. (2021). Systemic Inflammatory Biomarkers, Especially Fibrinogen to Albumin Ratio, Predict Prognosis in Patients with Pancreatic Cancer. Cancer Res. Treat..

[41] Tan Z., Zhang M., Han Q., Wen J., Luo K., Lin P., Zhang L., Yang H., Fu J. (2017). A novel blood tool of cancer prognosis in esophageal squamous cell carcinoma: The Fibrinogen/Albumin Ratio. J. Cancer.

[42] Howard R., Kanetsky P.A., Egan K.M. (2019). Exploring the prognostic value of the neutrophil-to-lymphocyte ratio in cancer. Sci. Rep..

[43] Ali S., Shahab S., Rauf M., Riaz S.K., Sheikh A.K., Tariq J., Gilani A., Gilani F. (2022). Neutrophil To Lymphocyte Ratio As A Predictor Of Severity In Colorectal Adenocarcinoma. J. Ayub Med. Coll. Abbottabad.

[44] Coffelt S.B., Wellenstein M.D., de Visser K.E. (2016). Neutrophils in cancer: Neutral no more. Nat. Rev. Cancer.

[45] Ocana A., Nieto-Jiménez C., Pandiella A., Templeton A.J. (2017). Neutrophils in cancer: Prognostic role and therapeutic strategies. Mol. Cancer.

[46] Steidl C., Lee T., Shah S.P., Farinha P., Han G., Nayar T., Delaney A., Jones S.J., Iqbal J., Weisenburger D.D. (2010). Tumor-associated macrophages and survival in classic Hodgkin’s lymphoma. N. Engl. J. Med..

[47] Ishigami S., Natsugoe S., Tokuda K., Nakajo A., Okumura H., Matsumoto M., Miyazono F., Hokita S., Aikou T. (2003). Tumor-associated macrophage (TAM) infiltration in gastric cancer. Anticancer Res..

[48] Hu G., Liu G., Ma J.Y., Hu R.J. (2018). Lymphocyte-to-monocyte ratio in esophageal squamous cell carcinoma prognosis. Clin. Chim. Acta..

[49] Condeelis J., Pollard J.W. (2006). Macrophages: Obligate partners for tumor cell migration, invasion, and metastasis. Cell.

[50] Hu R.J., Ma J.Y., Hu G. (2018). Lymphocyte-to-monocyte ratio in pancreatic cancer: Prognostic significance and meta-analysis. Clin. Chim. Acta..

[51] Rosenberg S.A. (2001). Progress in human tumour immunology and immunotherapy. Nature.

[52] Dunn G.P., Old L.J., Schreiber R.D. (2004). The immunobiology of cancer immunosurveillance and immunoediting. Immunity.

[53] Tan D., Fu Y., Tong W., Li F. (2018). Prognostic significance of lymphocyte to monocyte ratio in colorectal cancer: A meta-analysis. Int. J. Surg..

[54] Hoffmann T.K., Dworacki G., Tsukihiro T., Meidenbauer N., Gooding W., Johnson J.T., Whiteside T.L. (2002). Spontaneous apoptosis of circulating T lymphocytes in patients with head and neck cancer and its clinical importance. Clin. Cancer Res..

[55] Vayrynen J.P., Tuomisto A., Klintrup K., Makela J., Karttunen T.J., Makinen M.J. (2013). Detailed analysis of inflammatory cell infiltration in colorectal cancer. Br. J. Cancer.

[56] Gay L.J., Felding-Habermann B. (2011). Contribution of platelets to tumour metastasis. Nat. Rev. Cancer.

[57] Banks R.E., Forbes M.A., Kinsey S.E., Stanley A., Ingham E., Walters C., Selby P.J. (1998). Release of the angiogenic cytokine vascular endothelial growth factor (VEGF) from platelets: Significance for VEGF measurements and cancer biology. Br. J. Cancer.

[58] Ma J.Y., Ke L.C., Liu Q. (2018). The pretreatment platelet-to-lymphocyte ratio predicts clinical outcomes in patients with cervical cancer: A meta-analysis. Medicine.

[59] Mijic S., Dabrosin C. (2021). Platelet Activation In Situ in Breasts at High Risk of Cancer: Relationship with Mammographic Density and Estradiol. J. Clin. Endocrinol. Metab..

[60] Floris G., Richard F., Hamy A.S., Jongen L., Wildiers H., Ardui J., Punie K., Smeets A., Berteloot P., Vergote I. (2021). Body Mass Index and Tumor-Infiltrating Lymphocytes in Triple-Negative Breast Cancer. J. Natl. Cancer Inst..

[61] Gong Z., Xin R., Li L., Lv L., Wu X. (2022). Platelet-to-lymphocyte ratio associated with the clinicopathological features and prognostic value of breast cancer: A meta-analysis. Int. J. Biol. Markers.

[62] Palacios-Acedo A.L., Langiu M., Crescence L., Mège D., Dubois C., Panicot-Dubois L. (2022). Platelet and Cancer-Cell Interactions Modulate Cancer-Associated Thrombosis Risk in Different Cancer Types. Cancers.

[63] McMillan D.C. (2013). The systemic inflammation-based Glasgow Prognostic Score: A decade of experience in patients with cancer. Cancer Treat. Rev..

[64] Guthrie G.J., Charles K.A., Roxburgh C.S., Horgan P.G., McMillan D.C., Clarke S.J. (2013). The systemic inflammation-based neutrophil-lymphocyte ratio: Experience in patients with cancer. Crit. Rev. Oncol. Hematol..

[65] Qiang G., Liang C., Xiao F., Yu Q., Wen H., Song Z., Tian Y., Shi B., Guo Y., Liu D. (2016). Prognostic significance of platelet-to-lymphocyte ratio in non-small-cell lung cancer: A meta-analysis. Onco Targets Ther..

[66] Tan D., Fu Y., Su Q., Wang H. (2016). Prognostic role of platelet-lymphocyte ratio in colorectal cancer: A systematic review and meta-analysis. Medicine.

[67] Yodying H., Matsuda A., Miyashita M., Matsumoto S., Sakurazawa N., Yamada M., Uchida E. (2016). Prognostic Significance of Neutrophil-to-Lymphocyte Ratio and Platelet-to-Lymphocyte Ratio in Oncologic Outcomes of Esophageal Cancer: A Systematic Review and Meta-analysis. Ann. Surg. Oncol..

[68] Li B., Zhou P., Liu Y., Wei H., Yang X., Chen T., Xiao J. (2018). Platelet-to-lymphocyte ratio in advanced Cancer: Review and meta-analysis. Clin. Chim. Acta..

[69] Wang J., Zhou X., He Y., Chen X., Liu N., Ding Z., Li J. (2018). Prognostic role of platelet to lymphocyte ratio in prostate cancer: A meta-analysis. Medicine.

[70] Zhang Y., Zheng L., Quan L., Du L. (2021). Prognostic role of platelet-to-lymphocyte ratio in oral cancer: A meta-analysis. J. Oral. Pathol. Med..

[71] Zhang Y., Xing Z., Zhou K., Jiang S. (2021). The Predictive Role of Systemic Inflammation Response Index (SIRI) in the Prognosis of Stroke Patients. Clin. Interv. Aging..

[72] Zinellu A., Collu C., Nasser M., Paliogiannis P., Mellino S., Zinellu E., Traclet J., Ahmad K., Mangoni A.A., Carru C. (2021). The Aggregate Index of Systemic Inflammation (AISI): A Novel Prognostic Biomarker in Idiopathic Pulmonary Fibrosis. J. Clin. Med..

[73] Han K., Shi D., Yang L., Wang Z., Li Y., Gao F., Liu Y., Ma X., Zhou Y. (2022). Prognostic value of systemic inflammatory response index in patients with acute coronary syndrome undergoing percutaneous coronary intervention. Ann. Med..

[74] Yu C., Jiang H., Wang L., Jiang Z., Jin C. (2025). Baseline (derived) neutrophil-lymphocyte ratio associated with survival in gastroesophageal junction or gastric cancer treated with ICIs. Front. Oncol..

[75] Chen J.H., Zhai E.T., Yuan Y.J., Wu K.M., Xu J.B., Peng J.J., Chen C.Q., He Y.L., Cai S.R. (2017). Systemic immune-inflammation index for predicting prognosis of colorectal cancer. World J. Gastroenterol..

[76] Xie Q.K., Chen P., Hu W.M., Sun P., He W.Z., Jiang C., Kong P.F., Liu S.S., Chen H.T., Yang Y.Z. (2018). The systemic immune-inflammation index is an independent predictor of survival for metastatic colorectal cancer and its association with the lymphocytic response to the tumor. J. Transl. Med..

[77] Li J., Cao D., Huang Y., Xiong Q., Tan D., Liu L., Lin T., Wei Q. (2022). The Prognostic and Clinicopathological Significance of Systemic Immune-Inflammation Index in Bladder Cancer. Front. Immunol..

[78] Zhu M., Chen L., Kong X., Wang X., Fang Y., Li X., Wang J. (2022). The Systemic Inflammation Response Index as an Independent Predictor of Survival in Breast Cancer Patients: A Retrospective Study. Front. Mol. Biosci..

[79] Chen L., Kong X., Wang Z., Wang X., Fang Y., Wang J. (2020). Pretreatment Systemic Inflammation Response Index in Patients with Breast Cancer Treated with Neoadjuvant Chemotherapy as a Useful Prognostic Indicator. Cancer Manag. Res..

[80] Miyamoto K., Inai K., Takeuchi D., Shinohara T., Nakanishi T. (2015). Relationships among red cell distribution width, anemia, and interleukin-6 in adult congenital heart disease. Circ. J..

[81] Li N., Zhou H., Tang Q. (2017). Red Blood Cell Distribution Width: A Novel Predictive Indicator for Cardiovascular and Cerebrovascular Diseases. Dis. Markers.

[82] Patel R., English L., Liu W.K., Tree A.C., Ayres B., Watkin N., Pickering L.M., Afshar M. (2020). Red cell differential width (RDW) as a predictor of survival outcomes with palliative and adjuvant chemotherapy for metastatic penile cancer. Int. Urol. Nephrol..

[83] Chen W., Xin S., Xu B. (2022). Value Research of NLR, PLR, and RDW in Prognostic Assessment of Patients with Colorectal Cancer. J. Healthc. Eng..

[84] Cheng K.C., Lin Y.M., Liu C.C., Wu K.L., Lee K.C. (2022). High Red Cell Distribution Width Is Associated with Worse Prognosis in Early Colorectal Cancer after Curative Resection: A Propensity-Matched Analysis. Cancers.

[85] Wallerstein R.O. (1987). Laboratory evaluation of anemia. West. J. Med..

[86] Yildirim A., Duygulu M.E., Fidan E. (2024). CDK4/6 Inhibitor-Associated Mean Corpuscular Volume Change: A Potential Parameter for Predicting Survival in Metastatic Breast Cancer?. J. Coll. Physicians Surg. Pak..

[87] Wen Z.L., Zhou X., Xiao D.C. (2022). Is red blood cell distribution width a prognostic factor for colorectal cancer? A meta-analysis. Front. Surg..

[88] Palumbo J.S., Kombrinck K.W., Drew A.F., Grimes T.S., Kiser J.H., Degen J.L., Bugge T.H. (2000). Fibrinogen is an important determinant of the metastatic potential of circulating tumor cells. Blood.

[89] Im J.H., Fu W., Wang H., Bhatia S.K., Hammer D.A., Kowalska M.A., Muschel R.J. (2004). Coagulation facilitates tumor cell spreading in the pulmonary vasculature during early metastatic colony formation. Cancer Res..

[90] Black S., Kushner I., Samols D. (2004). C-reactive Protein. J. Biol. Chem..

[91] Allin K.H., Nordestgaard B.G. (2011). Elevated C-reactive protein in the diagnosis, prognosis, and cause of cancer. Crit. Rev. Clin. Lab. Sci..

[92] Valero C., Zanoni D.K., Pillai A., Ganly I., Morris L.G., Shah J.P., Wong R.J., Patel S.G. (2020). Host Factors Independently Associated With Prognosis in Patients With Oral Cavity Cancer. JAMA Otolaryngol..

[93] Sun J., Carrero J.J., Zagai U., Evans M., Ingre C., Pawitan Y., Fang F. (2020). Blood biomarkers and prognosis of amyotrophic lateral sclerosis. Eur. J. Neurol..

[94] McMillan D.C., Watson W.S., O’Gorman P., Preston T., Scott H.R., McArdle C.S. (2001). Albumin concentrations are primarily determined by the body cell mass and the systemic inflammatory response in cancer patients with weight loss. Nutr. Cancer..

[95] Shibutani M., Maeda K., Nagahara H., Iseki Y., Ikeya T., Hirakawa K. (2016). Prognostic Significance of the Preoperative Ratio of C-Reactive Protein to Albumin in Patients with Colorectal Cancer. Anticancer Res..

[96] Nakazaki H. (1992). Preoperative and postoperative cytokines in patients with cancer. Cancer.

[97] Ghezzi F., Cromi A., Siesto G., Giudici S., Serati M., Formenti G., Franchi M. (2010). Prognostic significance of preoperative plasma fibrinogen in endometrial cancer. Gynecol. Oncol..

[98] Luyendyk J.P., Schoenecker J.G., Flick M.J. (2019). The multifaceted role of fibrinogen in tissue injury and inflammation. Blood.

[99] Kattula S., Byrnes J.R., Wolberg A.S. (2017). Fibrinogen and Fibrin in Hemostasis and Thrombosis. Arterioscler. Thromb. Vasc. Biol..

[100] McMillan D.C. (2009). Systemic inflammation, nutritional status and survival in patients with cancer. Curr. Opin. Clin. Nutr. Metab. Care.

[101] Sohda M., Sakai M., Yamaguchi A., Watanabe T., Nakazawa N., Ubukata Y., Kuriyam K., Sano A., Yokobori T., Ogawa H. (2022). Pre-treatment CRP and Albumin Determines Prognosis for Unresectable Advanced Oesophageal Cancer. In Vivo.

[102] An Q., Liu W., Yang Y., Yang B. (2020). Preoperative fibrinogen-to-albumin ratio, a potential prognostic factor for patients with stage IB-IIA cervical cancer. BMC Cancer.

[103] Artigas A., Wernerman J., Arroyo V., Vincent J.L., Levy M. (2016). Role of albumin in diseases associated with severe systemic inflammation: Pathophysiologic and clinical evidence in sepsis and in decompensated cirrhosis. J. Crit. Care..

[104] Rothschild M.A., Oratz M., Schreiber S.S. (1988). Serum albumin. Hepatology.

[105] Sun F., Tan Y.A., Gao Q.F., Li S.Q., Zhang J., Chen Q.G., Jiang Y.H., Zhang L., Ying H.Q., Wang X.Z. (2019). Circulating fibrinogen to pre-albumin ratio is a promising biomarker for diagnosis of colorectal cancer. J. Clin. Lab. Anal..

[106] Li S., Zhang D., Zeng S., Wu T., Wang Y., Zhang H., Wang B., Hu X. (2021). Prognostic Value of Preoperative Albumin-to-Fibrinogen Ratio in Patients with Bladder Cancer. J. Cancer.

[107] Otowa Y., Nakamura T., Yamamoto M., Kanaji S., Matsuda Y., Matsuda T., Oshikiri T., Sumi Y., Suzuki S., Kakeji Y. (2017). C-reactive protein to albumin ratio is a prognostic factor for patients with Stage II/III esophageal squamous cell cancer. Dis. Esophagus.

[108] Tamagawa H., Aoyama T., Tamagawa A., Komori K., Maezawa Y., Kano K., Murakawa M., Atsumi Y., Hara K., Kazama K. (2020). Influence of the Preoperative C-Reactive Protein-to-Albumin Ratio on Survival and Recurrence in Patients With Esophageal Cancer. Anticancer Res..

[109] Liu Y., Chen S., Zheng C., Ding M., Zhang L., Wang L., Xie M., Zhou J. (2017). The prognostic value of the preoperative c-reactive protein/albumin ratio in ovarian cancer. BMC Cancer.

